# Methoxy-Functionalized Conjugated Self-Assembled Monolayers as a Hole-Selective Contact for Inverted Perovskite Solar Cells

**DOI:** 10.1021/acsami.6c04007

**Published:** 2026-05-18

**Authors:** Shamim Ahmmed, Md. Abdul Karim, Md. Emrul Kayesh, Masatoshi Yanagida, Yasuhiro Shirai, Kensuke Kojima, Akito Hayashi, Jun Azuma, Kiyoto Matsuishi, Ashraful Islam

**Affiliations:** † Photovoltaic Materials Group, Center for Green Research on Energy and Environmental Materials, 52747 National Institute for Materials Science (NIMS), 1-2-1 Sengen, Tsukuba, Ibaraki 305-0047, Japan; ‡ Photovoltaic Materials Group, Center for Green Research on Energy and Environmental Materials, National Institute for Materials Science (NIMS), 1-1 Namiki, Tsukuba, Ibaraki 305-0044, Japan; § Research & Development Department, Corporate Photoreceptor Division, 730745 KYOCERA Document Solutions Inc, 1-2-28 Tamatsukuri, Chuo-ku, Osaka 540-8585, Japan; ∥ Institute of Pure and Applied Sciences, 13121 University of Tsukuba, 1-1-1 Tennodai, Tsukuba, 305-8573 Ibaraki, Japan

**Keywords:** self-assembled monolayer, methoxy group, conjugated linker, triphenylamine, perovskite solar cell

## Abstract

In recent years, significant progress in inverted (p-i-n) perovskite solar cells (PSCs) has been observed, primarily due to the development of innovative self-assembled monolayer (SAM) materials for the hole transport layer (HTL). SAM materials have gained more popularity over the conventional polymer HTL material poly­[bis­(4-phenyl)­(2,4,6-trimethylphenyl)­amine] (PTAA) because of their efficient hole transport and ability to passivate interfaces. Conjugated SAMs are considered as a good choice for PSCs because of their electrical and photostability. In this research, methoxy-functionalized conjugated SAMs (C21 and C22) were synthesized and studied for inverted Pb-based PSCs. Good coverage of the perovskite layer was observed on the C21 and C22 SAMs. The SAM molecules on fluorine-doped tin oxide (FTO) tuned the work function (*W*_F_) of the FTO with the number of methoxy groups in the SAM molecule. The methoxy group positioned at ortho- and para-positions was found favorable for the growth of good perovskite layers on the SAM surface. As a result, the C22 SAM with the Pb-based triple cation perovskite in the inverted PSC exhibited a champion power conversion efficiency (PCE) of 21.58%. These findings suggest that conjugated SAM functionalized with methoxy groups at the ortho- and para-positions are promising for the further development of inverted PSCs.

## Graphical abstract

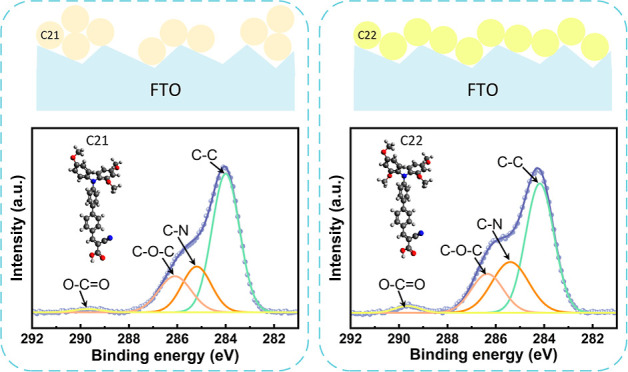

## 1 Introduction

In the past few years, significant work has been dedicated to developing novel perovskite materials and improving device structures, leading to an increase in the power conversion efficiency (PCE) of perovskite solar cells (PSCs) from 3.8% to over 27%.^1,2^ In the highly efficient PSCs, triple cation perovskites (CsFAMA) are primarily utilized due to their greater thermal and phase stability in comparison to single cation perovskites.^3,4^ The PSC features two types of configurations: the conventional (n-i-p) and inverted (p-i-n) structures. Currently, the PCE of PSCs in both n-i-p and p-i-n configurations is almost the same.^5^ Despite this, the rise in the popularity of the inverted structure PSCs has been growing due to their distinct benefits, such as processing at low temperatures, reduced hysteresis, enhanced stability, and compatibility with perovskite tandem solar cells.^2,6–10^

The selection or design of the hole transport layer (HTL) is particularly important to the performance and stability of the inverted PSCs, since it greatly affects the quality of the perovskite film.^7,11,12^ Additionally, because it is located at the light incident side of this device architecture, it should be quite robust. In the inverted PSCs, various materials have been studied as an HTL, including nickel oxide (NiO_*x*_),^13^ self-assembled monolayers (SAMs),^14^ poly­(3,4-ethylenedioxythiophene) polystyrenesulfonate (PEDOT:PSS),^15^ and poly [bis (4-phenyl) (2,4,6-trimethylphenyl) amine] (PTAA).^16^ NiO_*x*_ is being investigated as an HTL in inverted PSCs due to its wide band gap (3.6–4.0 eV), excellent hole mobility, low cost, and easy solution processability.^13,17^ Nevertheless, the deprotonation reaction between the perovskite halides and Ni^3+^ is accelerated by the presence of Ni^3+^ at the HTL/perovskite interface, lowering the stability and performance of the device.^13,18^ PEDOT:PSS deteriorates the perovskite layer at the HTL/perovskite interface due to its acidic nature, hindering the long-term stability of the PSC.^19,20^ PTAA experiences inadequate wettability, leading to the insufficient coverage of the perovskite layer.^21,22^ Furthermore, PSCs utilizing the PTAA HTL also experience problems with a low fill factor.^11,23^

Among the diverse kinds of HTLs used in the inverted-structured PSCs, the anchoring-based SAM approach has recently shown superior performance compared to the conventional HTLs.^24,25^ The significance of SAM in PSCs stems from their minimal material usage, adjustable work functions (*W*_F_), favorable energy-level alignment, high light transparency, and straightforward deposition process.^25^ The molecular configuration of SAM often features with a hole-transporting terminal group which could be decorated with the functional group, an anchoring group that can chemically adsorb on the surface of transparent conductive oxides (like fluorine-doped tin oxide (FTO) or indium tin oxide), along with a linker that facilitates self-assembly between these components.^26^ With significant efforts, diverse SAMs have been developed in recent years by tailoring the terminal, linker, and anchoring groups, enabling precise tuning of energy level alignment, suppression of interfacial defects, and enhancement of the stability and efficiency of PSCs.^26–28^

In this research, we have designed and synthesized methoxy-functionalized triphenylamine-based conjugated SAMs and introduced them into the triple-cation Pb-based inverted PSCs. Carbazole and its derivatives are commonly utilized as terminal group materials for designing SAMs. Nevertheless, we designed triphenylamine-based SAMs because of their superior thermal stability and better regulation of electrical properties than those of carbazole-based SAMs.^29,30^ The nonconjugated alkyl linkers diminish the stability of the electron-rich arylamine groups and impair the carrier transport efficiency because of the localization of electrons.^31^ We used the conjugated linkers due to their ability to enhance the stability of the electron-rich arylamines by promoting effective delocalization of electrons.^29,32,33^ Most of the SAMs are designed with the terminal group decorated with different functional groups (Table S1) due to their significant impact on the features of SAM as well as the growth of the perovskite layer on SAM. Recently, Zhang and co-workers have reported that methoxy as a functional group in both conjugated and nonconjugated SAMs improves the capability of hole extraction and helps in good wettability of the perovskite precursor on SAM due to the enhanced surface polarity.^33^ Moreover, Aktas and co-workers have observed that the position of the methoxy group in the arylamine regulates its electron-donating or electron-withdrawing features as well as steric hindrance.^34^ So far, various methoxy-functionalized SAMs like MeO-2PACz,^14^ MeO-PhPACz,^35^ MeOF-4SHCz,^36^ MeO-4PADBC,^37^ MPA-CPA,^38^ MeOF-NaPACz,^33^ and MPA-Ph–CA^29^ have been studied for the inverted PSCs. However, there have been only a few studies focusing on triphenylamine-based conjugated SAMs, and there is a lack of comprehensive research on how the number and position of methoxy groups influence these SAMs and the PSCs.

Here, we synthesized the triphenylamine-based conjugated SAMs functionalized with different numbers of methoxy groups in different positions of the triphenylamine and studied their role on the growth of the perovskite layer and performance of the inverted Pb-based PSCs. The perovskite layer growth was found to be better on the conjugated SAM functionalized with the methoxy group in ortho- and para-positions (named C22), which showed a superior performance and stability in the PSCs. The C22 SAM-based PSCs showed an optimized PCE of 21.58% with good stability under continuous sunlight.

## 2 Experimental Section

### 2.1 Materials

Most reagents used for the synthesis of SAMs were purchased from the Tokyo Chemical Industry Co., Ltd., unless otherwise noted. Sodium *tert*-butoxide was purchased from the Kanto Chemical Co., Inc. 1-Bromo-4-iodobenzene and *p*-formylphenylboronic acid were purchased from the Angene International Limited. Piperidine and triphenylphosphine were purchased from Nacalai Tesque, Inc. All solvents used for the synthesis of SAMs, except tetrahydrofuran (THF), were purchased from Daishin Chemical Co. Ltd.; tetrahydrofuran (dehydrated/additive-free) was purchased from Fujifilm Wako Pure Chemical Corporation.

Materials for device fabrication were purchased as follows: cesium iodide (CsI, 99%), toluene (≥99.8%), chlorobenzene (CB, 99.5%), and isopropanol (IPA, ≥99.8%) were purchased from the Sigma-Aldrich. Methylammonium bromide (MABr, 98%), formamidinium iodide (FAI, >98%), lead­(II) iodide (PbI_2_, 99.99%), and lead­(II) bromide (PbBr_2_, 98%) were purchased from the Tokyo Chemical Industry Co., Ltd. Dimethyl sulfoxide (DMSO) and *N*,*N*-dimethylformamide (DMF) were purchased from Fujifilm Wako Pure Chemical Corporation. All materials were used as received without further purification.

### 2.2 Synthesis of C21 and C22 SAMs

The synthesis route of the C21 and C22 SAMs is presented in Schematic S1 and discussed in the Supporting Information.

### 2.3 Device Fabrication

The precleaned FTO substrates were treated by UV–O_3_ for 30 min for all samples. For C21 and C22 SAM deposition, a solution of 0.3 mg/mL was prepared in the ethanol solvent by stirring for 1 h. Then, the SAM solution was spin coated on the FTO by spinning at 5000 rpm for 50 s. Then, the samples were annealed at 100 °C for 10 min in a N_2_ filled glovebox. A 1.2 M perovskite (Cs_0.05_FA_0.80_MA_0.15_PbI_2.75_Br_0.25_ with 9% excess PbI_2_) solution was prepared in DMF and DMSO mixed solvents (4:1) by stirring for 3 h. The perovskite solution was spin coated on the SAM surface at a slope of 5 s, 1000 rpm for 10 s and 3 s, 4000 rpm for 20 and 2 s by using the one-step antisolvent (200 μL chlorobenzene was dropped during the 20th second) technique followed by annealing at 150 °C for 20 min. Then, 0.5 mg/mL EDAI_2_ solution in a 1:1 toluene and isopropanol mixed solvent was dynamically spin coated on the perovskite surface by spinning at 3000 rpm for 40s and annealed at 70 °C for 5 min. Then, the samples were moved into a vacuum chamber and sequentially deposited C_60_ (30 nm), BCP (7 nm), and Ag (100 nm) electrodes of area of 0.09 cm^2^ by the thermal vacuum evaporation technique at a pressure of <10^–4^ Pa.

### 2.4 Characterization

^1^H NMR spectra were recorded on a JEOL JNM-ECA600 (600 MHz) spectrometer. The surface roughness of the samples was monitored by a scanning probe microscope (SPM/AFM, Bruker’s Dimension Icon). The X-ray diffraction property of the perovskite samples was investigated by a MiniFlex 600 (Rigaku Co., Japan). The perovskite top surface scanning electron microscopy (SEM) images were obtained from the JSM-6500F field emission. Ultraviolet photoemission spectroscopy (UPS) and X-ray photoelectron spectroscopy (XPS) measurements were accomplished by ULVAC-PHI (PHI VersaProbe 4). For the current–voltage (*J*–*V*) characteristics analysis, a AM 1.5 G solar simulator (WXS-155S-10, Wacom Denso Co., Japan) was used and the analysis was performed under ambient conditions. The incident photon-to-current conversion efficiency (IPCE) spectra were observed by the CEP-2000BX (Bunkoukeiki Co. Ltd., Japan). The PAIOS system was used to study the Nyquist plot of the PSCs.

### 2.5 Density Functional Theory Calculation

DFT calculations were carried out to gain insights into the molecular structures of the SAMs. ORCA version 6.1.1 software was used for the DFT calculation. The single-molecule structure of SAMs was optimized, and calculations of the electrostatic surface potential and dipole moment were performed using the DFT theory of B3LYP with a basic set of def2-TZVP.

## 3 Results and Discussion

In this study, we designed and synthesized the methoxy-functionalized conjugated C21 and C22 SAMs. The structures of the synthesized molecules were confirmed by the ^1^H NMR and direct analyses in real-time mass spectrometry (DART-MS) (as shown in Figure S1–S4). NMR chemical shifts are reported in ppm relative to an internal standard, TMS (δ = 0.00 ppm). The residual solvent peaks from dimethyl sulfoxide (DMSO; δ = 2.49 ppm) and water (δ = 3.3–3.4 ppm) were also observed. The synthesized C21 and C22 SAMs were introduced in the inverted PSCs. The molecular structure and performance of the different nonconjugated^39–42^ and conjugated SAMs^29^ along with our synthesized C21 and C22 SAMs are illustrated in Figure 1.

**Figure 1:**
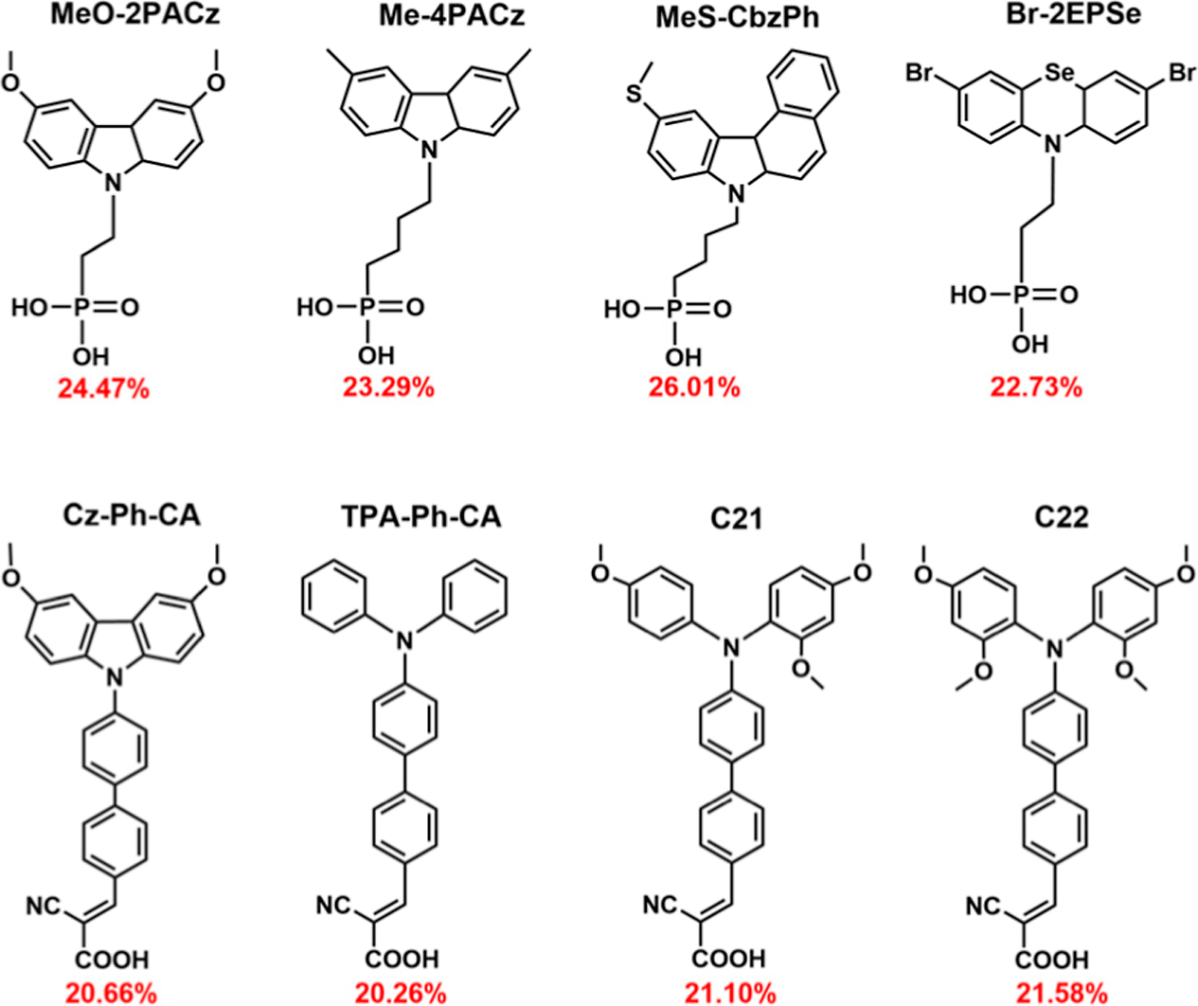
Chemical structures and performance of the reported and our synthesized (C21 and C22) SAMs.

The electrostatic surface potential (ESP) and dipole moment of the C21 and C22 SAMs were calculated by using the density functional theory (DFT) method. Figure 2a illustrates the ESP map of the C21 and C22 SAMs. Dipole moment values of 10.1 and 12.3 D were obtained for the C21 and C22 SAMs, respectively. The methoxy groups in the C22 SAM produce more negative potential around the triphenylamine due to the higher number of methoxy groups at the ortho- and para-positions in the C22 SAMs. As a result, C22 exhibits a stronger dipole moment. Figure 2b displays the schematic illustration of the possible interfacial interactions at the fluorine-doped tin oxide (FTO)/SAM/perovskite interface. The methoxy groups at the para-position of the SAMs are close to the perovskite crystal; these may interact with the uncoordinated Pb^2+^ and passivate the defects at the SAM/perovskite interface.

**Figure 2:**
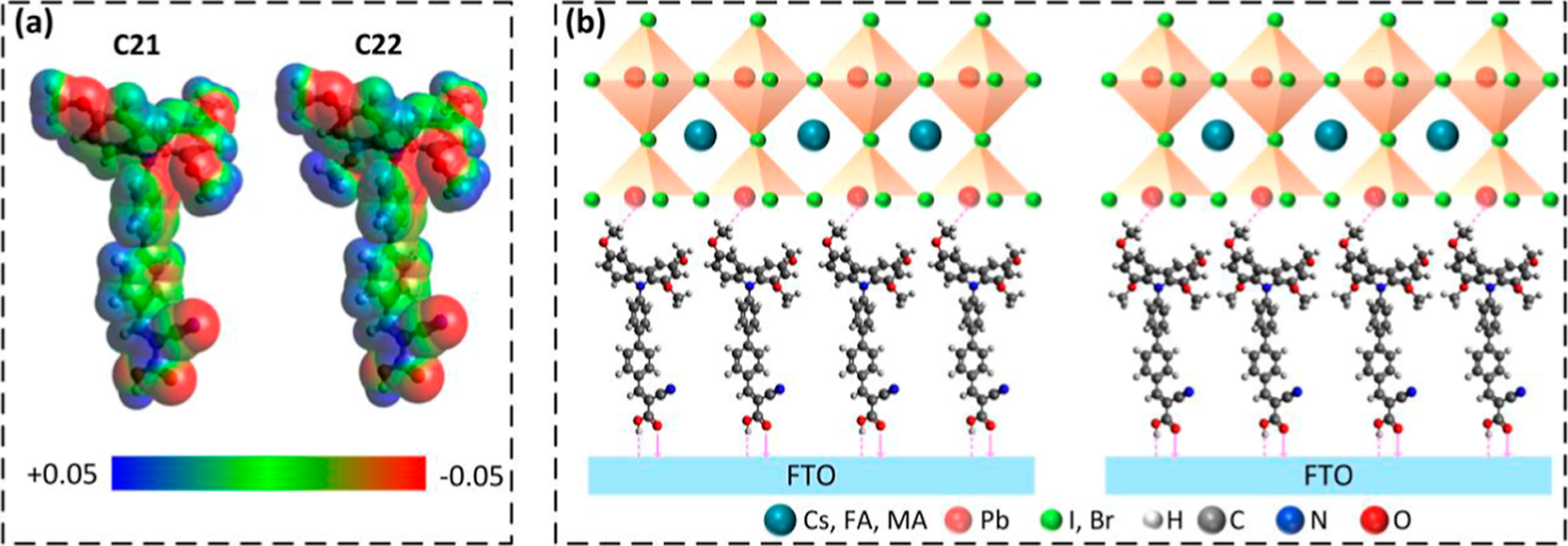
(a) Electrostatic surface potential mapping of C21 and C22 SAMs. (b) Schematic illustration of interfacial interactions at the FTO/SAM/perovskite interface.

The synthesized C21 and C22 SAMs were introduced in the inverted Pb-based PSCs as an HTL. Since the PSCs were fabricated on the FTO-coated substrates, the compositions of C21 and C22 SAMs deposited on FTO were analyzed by the X-ray photoelectron spectrometry (XPS). The anchoring of C21 and C22 SAMs on FTO can be identified from the C 1s XPS spectra. The C 1s spectra were deconvoluted into four peaks assigned to C–C, O–C=O, C–O–C, and C–N for both C21 and C22 SAMs (shown in Figure 3a,b and Table S2). The peaks assigned as O–C=O and C–N confirmed that the C21 and C22 SAMs were successfully anchored on the FTO surface. Besides, it is obvious from Figure 3c that the area of O–C=O and C–N peaks of C22 SAM is higher than the C21 SAM, which indicates the formation of the denser film of C22 SAM on the FTO. The symmetric structure and the larger number of methoxy groups at the ortho-position in the C22 SAM compared with the C21 SAM increase steric hindrance in the C22 SAM, which may suppress the aggregation of SAM in solution and form a dense and conformal SAM layer on the rough FTO surface.^43^ ^,^ ^44^ The dense film of hole-selective monolayers is crucial for suppressing the leakage current in the device.^45^ ^,^ ^46^

**Figure 3:**
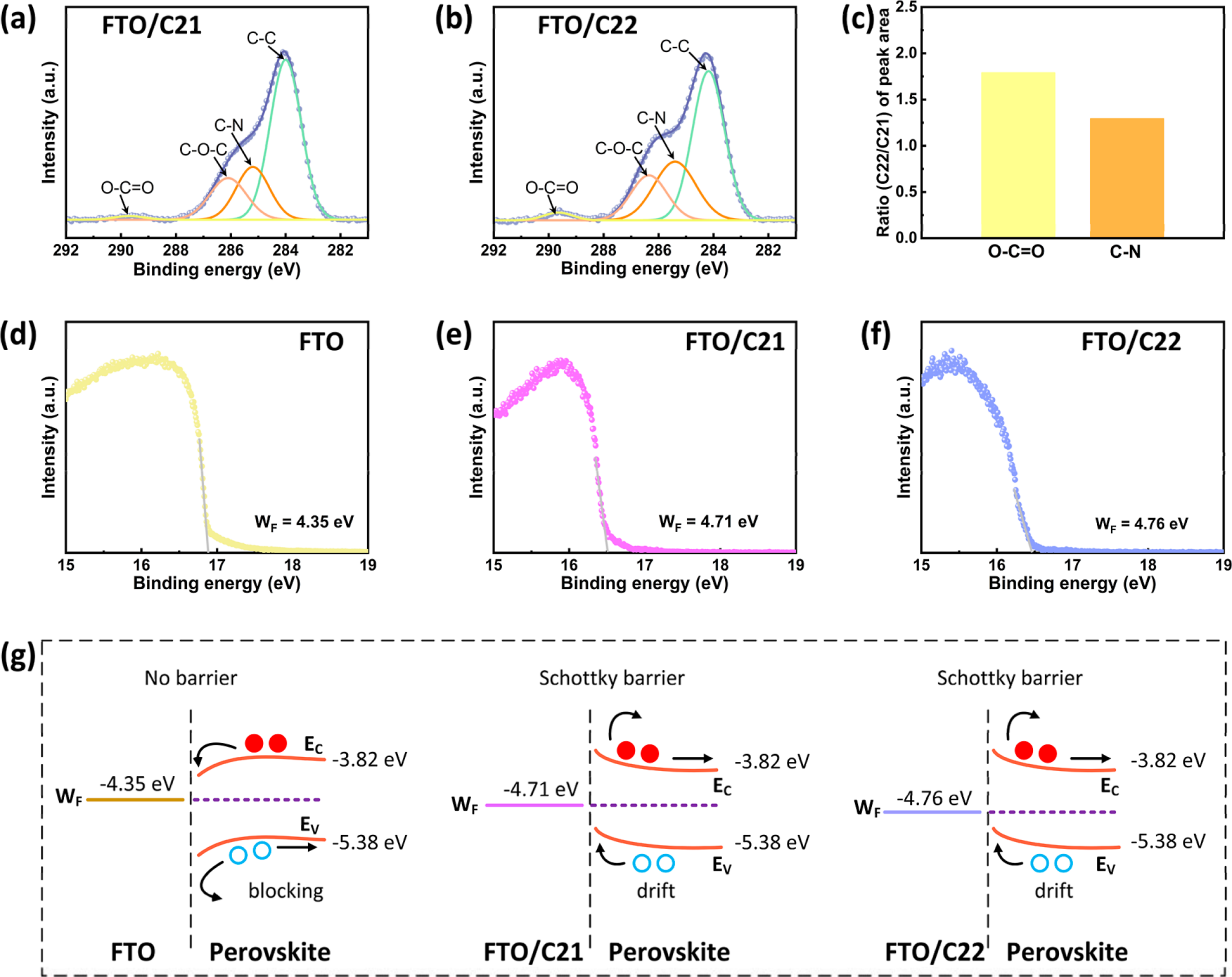
XPS spectra of C 1s of the (a) C21 and (b) C22 monolayer films on the FTO. (c) Peak area ratios (C22/C21) of O–C=O and C–N bonds extracted from the C 1s XPS spectra. UPS spectra of the secondary electron cutoff regions of the (d) FTO, (e) FTO/C21, and (f) FTO/C22. (g) Energy diagrams of the FTO/perovskite, FTO/C21/perovskite, and FTO/C22/perovskite interfaces.

The energy alignment between the perovskite absorber layer and the hole-selective layer determines the hole transport efficiency and device performance. The ultraviolet photoelectron spectroscopy (UPS) analysis was conducted on the FTO, C21, and C22 deposited on FTO substrate (shown in Figures 3d–f and S5). The secondary electron cutoff regions of UPS spectra of the samples are displayed in Figure 3d–f. It is obvious from Figure 3d–f that the work function (*W*_F_) of the FTO was increased after deposition of C21 and C22 SAMs on the FTO, suggesting the formation of a strong dipole at the FTO/SAM interface.

The energy band diagrams of the FTO/perovskite, FTO/C21/perovskite, and FTO/C22/perovskite interfaces are illustrated in Figure 3g (perovskite energy alignment was adopted from our previous study^47^). The barrier-free contact for electrons was formed at the FTO/perovskite interface due to the small energy offset between the *W*_F_ of FTO (−4.35 eV) and the conduction band minimum (*E*_C_) of the perovskite layer. Meanwhile, the larger energy offset between the *W*_F_ of FTO and the valence band maximum (*E*_V_) of the perovskite layer blocked the flow of holes from the perovskite to the FTO electrode. After deposition of C21 and C22 SAMs on the FTO, the increased *W*_F_ values of the FTO/C21 (−4.71 eV) and FTO/C22 (−4.76 eV) resulted in the upward band bending in the perovskite upon contact with the FTO/C21 and FTO/C22. The hole transfer from the perovskite to the FTO electrode was accelerated by upward band bending in the perovskite. Meanwhile, the upward band bending in the perovskite blocked the electron flow from the perovskite to the FTO electrode, which could reduce the recombination rate and enhance the device performance.^42^ Moreover, the barrier energy for holes at the FTO/C22/perovskite interface is slightly lower compared to the FTO/C21/perovskite interface, which might be due to the higher dipole moment of C22 compared to C21.^48^ Therefore, the FTO/C22/perovskite interface would be favorable to reduce the *V*_OC_ loss of the PSCs.^49^

The surface properties of the C21 and C22 SAMs deposited on the FTO were studied by using the atomic force microscopy (AFM) and water contact angle (WCA) measurements. Figures 4a,b and S6 display the AFM, WCA, and optical images of the different samples. From Figure 4a,b, the average roughness (*R*_av_) values of 24.8 and 24.2 nm were observed from the C21 and C22 deposited on the FTO substrate. The WCA values of 22.0° and 24.7° were observed from the C21 and C22 deposited on the FTO substrate. The slightly higher WCA observed for C22 compared to C21 may be attributed to the more uniform and better coverage of C22 on the FTO surface. The optical image (Figure S6) depicts that the perovskite on C21 and C22 SAMs exhibited very good coverage. The methoxy group in C21 and C22 SAM might help in the good coverage of the perovskite layer on C21 and C22 SAMs.^38,41^

**Figure 4:**
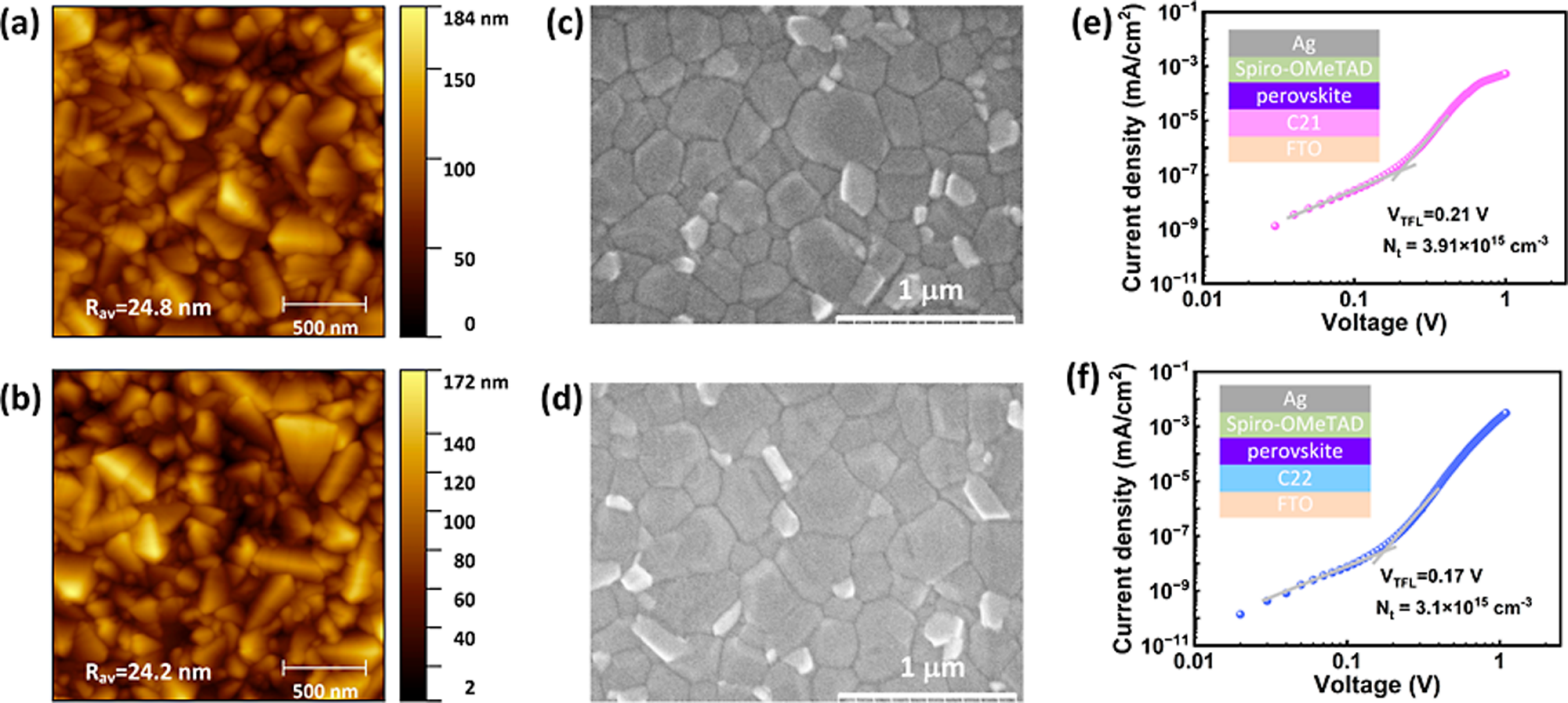
AFM images of (a) C21 and (b) C22 SAMs deposited on the FTO. Perovskite surface SEM images on (c) C21 and (d) C22 SAMs. Dark *J*–*V* characteristics of (e) C21 and (f) C22-based hole only devices.

The X-ray diffraction (XRD) analysis was conducted to analyze the crystallographic properties of the perovskite deposited on C21 and C22 samples. The XRD analysis data are illustrated in Figure S7. The crystallographic properties were evaluated using the Halder–Wagner plot depicted in Figure S8 and listed in Table S3. It is obvious from Table S3 that the crystallinity of the perovskite deposited on C22 SAM was slightly higher than the perovskite deposited on C21 SAM. In addition, a slightly lower lattice strain was observed from the perovskite deposited on C22 than the perovskite deposited on C21. The good crystallinity and low lattice strain of perovskites facilitate good carrier mobility which is advantageous for device performance.^50–52^

The surface morphology of the perovskite deposited on C21 and C22 SAMs was investigated by employing scanning electron microscopy (SEM). Figure 4c,d presents the top surface SEM images of the perovskite deposited on C21 and C22 SAMs. The grain size distributions (Figure S9) of different perovskite films were estimated from the SEM images. The perovskite grain size is slightly larger on the C22 SAM than on the C21 SAM, which might be due to the presence of methoxy groups at the ortho- and para-positions in the symmetric structure of the C22 SAM.^34^ The perovskite on C21 and C22 SAMs consists of pinhole-free and compact grains. The good coverage of the perovskite on C21 and C22 inhibited the formation of voids in the buried perovskite interface, leading to compact grains of the perovskite.^53^

To get more insights about the defect features, the hole-only devices (HODs) of the perovskite were fabricated on the C21 and C22 SAMs along with the spiro-OMeTAD as a common HTL. The current density versus voltage (*J*–*V*) measurements of the HODs were conducted under dark conditions and illustrated in Figure 4e,f. From Figure 4e,f, the trap-filling limit voltages (*V*_TFL_) of 0.21 and 0.17 V were observed for the C21- and C22-based HODs, respectively. The *V*_TFL_ was determined from the intersect of the trap-filling and ohmic conduction regions. The defect density (*N*_t_) of HODs can be calculated from the values of *V*_TFL_ using the following eq (eq 1).

1 $$N_{t}=\frac{2\varepsilon \varepsilon_{o}V_{TFL}}{eL^{2}}$$

where, ε is the perovskite dielectric constant, ε_o_ is the vacuum dielectric constant, *V*_TFL_ is the trap-filled limit voltage, *L* is the perovskite thickness, and *e* is the electron elementary charge. The *N*_t_ values of 3.91 × 10^15^ and 3.10 × 10^15^ cm^–3^ were calculated for the C21- and C22-based HODs, respectively. The C22-based HOD showed a lower *N*_t_ value, indicating the lower buried interface defects at the C22/perovskite interface. The good coverage of C22 SAM on the FTO as well as the perovskite layer on C22 SAM might be the reason for the lower *N*_t_ value of the C22-based HOD. The suppression of buried interface defects at the C22/perovskite interface would be favorable for the good device performance of the C22 SAM-based PSCs.^11,38^

A schematic of the inverted PSC is illustrated in Figure 5a. In order to gain insights into the carrier transport characteristics of the C21 and C22 SAM-based inverted PSCs, electrochemical impedance spectroscopy (EIS) was employed. The EIS measurement was conducted at a bias voltage of 0.5 V with 40 mV amplitude to observe the Nyquist plot. In the Nyquist plot, the low-frequency region corresponds to the recombination resistance (*R*_rec_). As shown in Figure 5b, PSCs based on the C22 SAM demonstrated a higher *R*_rec_ compared to the PSCs with the C21 SAM. The higher *R*_rec_ value signifies a lower interface recombination rate and improved carrier transport efficiency in the C22-based PSCs.

**Figure 5:**
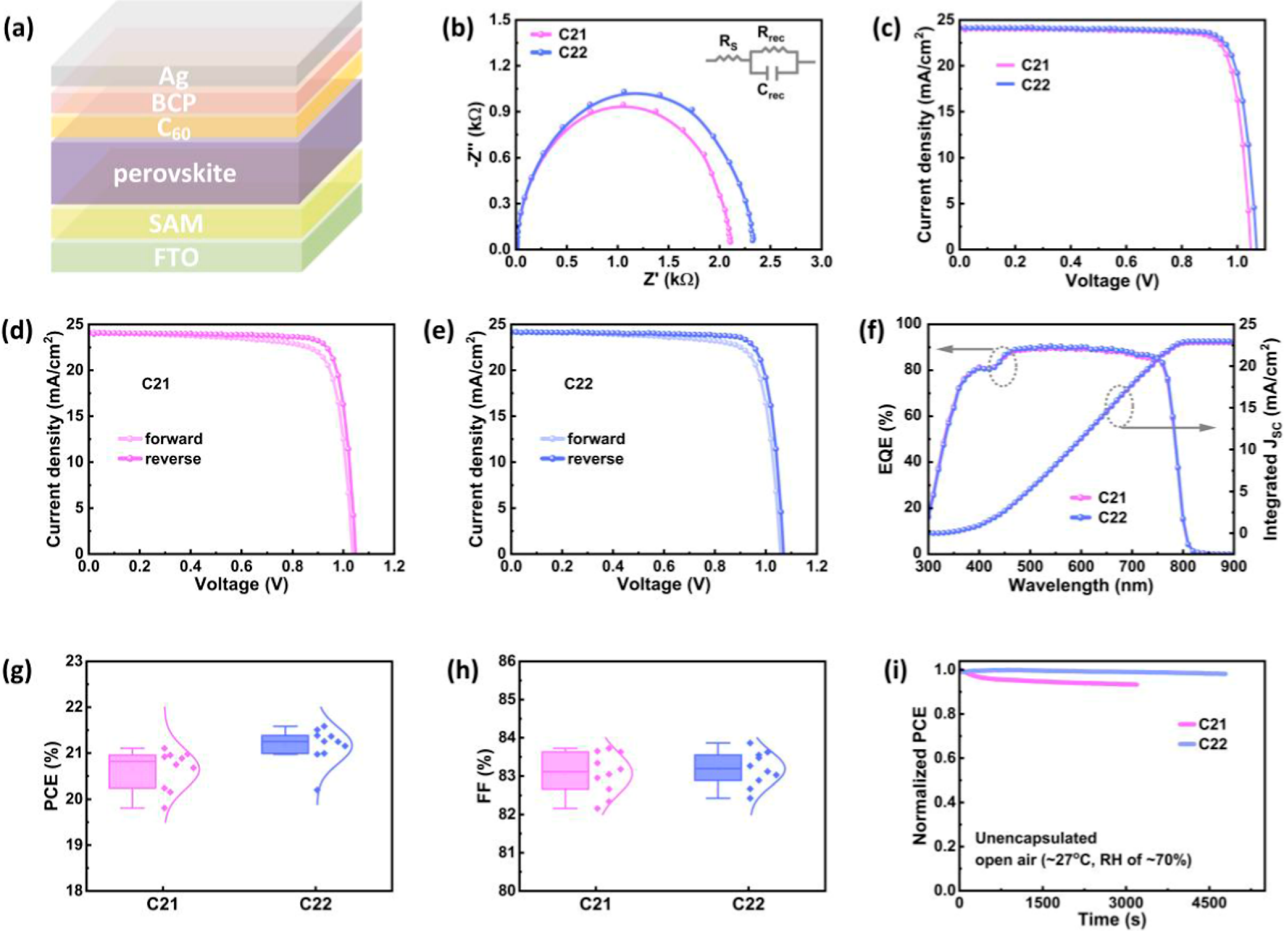
(a) Schematic diagram of the inverted PSCs. (b) Nyquist plots of the C21 and C22 SAM-based PSCs. (c) *J*–*V* characteristics of the C21 and C22 SAM-based PSCs under a reverse scan. *J*–*V* characteristics of the (d) C21 and (e) C22 SAM-based PSCs. (f) EQE and corresponding integrated *J*_SC_ of the C21 and C22 SAM-based PSCs. Statistical distributions of the (g) PCE and (h) FF of the C21 and C22 SAM-based PSCs under a reverse scan. (i) MPPT data of the unencapsulated PSCs with C21 and C22 SAMs (at ∼27 °C and RH of ∼70%).

The photovoltaic performance of the inverted PSCs using the different SAMs was studied and depicted in Figure 5c–e. The maximum performance of the devices was obtained under a reverse scan. Photovoltaic performance of the different HTL-based PSCs is listed in Table 1. The photovoltaic parameters of the PTAA-based device were adopted from our previous study.^11^ The PTAA HTL-based device was fabricated using the same conditions as the C21 and C22 SAM-based devices. The C21 and C22 SAM-based PSCs showed a better performance compared to the PTAA HTL-based PSC. Meanwhile, the C22 SAM-based PSCs showed the highest PCE of 21.58% with *V*_OC_, *J*_SC_, and FF of 1.07 V, 24.12 mA/cm^2^, and 83.63%, respectively. Meanwhile, the C21 SAM-based PSC displayed the optimized PCE of 21.1% with *V*_OC_, FF, and *J*_SC_ of 1.05 V, 83.62%, and 24.06 mA/cm^2^, respectively. The *J*_SC_ values measured from the *J*–*V* curves are consistent with the integrated *J*_SC_ values of the C21 (22.82 mA/cm^2^) and C22 (22.98 mA/cm^2^) SAM-based PSCs measured from the external quantum efficiency (EQE) spectra illustrated in Figure 5f.^54^ The C22 SAM-based PSCs displayed marginally better performance than those based on C21 SAM. In particular, the *V*_OC_ and *J*_SC_ were observed slightly higher from the C22 SAM-based PSCs. The enhanced *V*_OC_ of C22 SAM-based PSCs might be due to the lower Δ*E*_h_ at the FTO/C22/perovskite interface and fewer interface defects at the C22/perovskite interface. Besides, a larger grain size of the perovskite on C22 contributed to a slight improvement in *J*_SC_.

**Table 1:** Photovoltaic Performance of the PTAA,^11^ C21, and C22 HTL-Based PSCs

SAM	scan	*V*_OC_ (V)	*J*_SC_ (mA/cm^2^)	FF (%)	PCE (%)	HI
PTAA	forward	1.06	23.21	73.77	18.20	0.061
	reverse	1.08	22.49	79.23	19.36	
C21	forward	1.04	24.19	78.93	19.79	0.062
	reverse	1.05	24.06	83.62	21.10	
C22	forward	1.06	24.28	79.40	20.39	0.055
	reverse	1.07	24.12	83.63	21.58	

The hysteresis index (HI) of the C21 and C22 SAM-based PSCs was calculated by using the following equation (eq 2).

2 $$HI=\frac{{PCE}_{r}-{PCE}_{f}}{{PCE}_{r}}$$

where, PCE_r_ is the PCE at the reverse scan and PCE_f_ is the PCE at the forward scan. The HI values of 0.062 and 0.055 were determined for the C21 and C22 SAM-based PSCs, respectively. The C22 SAM-based PSCs showed the lower HI, which might be due to the good coverage of C22 on the FTO and lower defects at the C22/perovskite interface.

The statistical distributions of the photovoltaic parameters for different SAM-based PSCs are shown in Figures 5g,h and S10. The PCE of the C22 SAM-based PSCs showed a lower standard deviation than the C21 SAM-based PSCs. This suggests that the C22 SAM-based PSCs are more reproducible. The good coverage of the C22 SAM on the FTO surface and the perovskite layer on the C22 SAM may contribute to the better reproducibility of the C22 SAM-based PSCs. The maximum power point tracking (MPPT) of the unencapsulated C21 and C22 SAM-based PSCs was studied under open air conditions, a temperature of approximately 27 °C, and relative humidity (RH) of around 70%. The MPPT data of the C21 and C22 SAM-based PSCs are illustrated in Figure 5i. It is obvious from Figure 5i that the C22 SAM-based PSCs showed better stability than the C21 SAM-based PSCs. The better stability of the C22 SAM-based PSCs might be attributed to the formation of good quality perovskite layer on the C22 and the lower density of interface defects at the C22/perovskite interface. These findings indicate that conjugated SAMs functionalized with methoxy groups at ortho- and para-positions are advantageous for the device performance and stability.

## 4 Conclusions

In this study, methoxy-functionalized conjugated SAMs (C21 and C22) were synthesized and investigated for the inverted Pb-based PSCs. The perovskite layer exhibited good coverage on both C21 and C22 SAMs. Nevertheless, the presence of a methoxy group at the ortho- and para-positions was identified as superior for the growth of high-quality perovskite films on the conjugated SAM (C22) surface. As a result, a maximum PCE of 21.58% was observed for the C22 SAM-based inverted PSCs. These results reveal that the C22 SAM could be a potential candidate for the fabrication of efficient and stable inverted PSCs.

## Supplementary Material



## Notes

Synthesis route of SAMs, ^1^H NMR spectra, DART-MS spectra, XRD analysis, Halder–Wagner plots, grain size distribution plot, and photovoltaic parameters (PDF)
