# Supplementary material for: Methoxy-Functionalized Conjugated Self-Assembled Monolayers as a Hole-Selective Contact for Inverted Perovskite Solar Cells
Source: ACS Appl Mater Interfaces. 2026 May 18;18(20):28728–36. doi: 10.1021/acsami.6c04007 (PMC13220230; doi:10.1021/acsami.6c04007)
Supplement: Supplementary file 1 [file am6c04007_si_001.pdf]

Supporting Information

Methoxy-Functionalized Conjugated Self-Assembled Monolayer as Hole-Selective Contact for Inverted Perovskite Solar Cells

Shamim Ahmmed[1], Md. Abdul Karim[1], Md. Emrul Kayesh[1], Masatoshi Yanagida[2], Yasuhiro Shirai [2], Kensuke Kojima[3], Akito Hayashi[3], Jun Azuma[3], Kiyoto Matsuishi[4], Ashraful Islam[1*]

[1]Photovoltaic Materials Group, Center for Green Research on Energy and Environmental Materials, National Institute for Materials Science (NIMS), 1-2-1 Sengen, Tsukuba, Ibaraki 305-0047, Japan

[2]Photovoltaic Materials Group, Center for Green Research on Energy and Environmental Materials, National Institute for Materials Science (NIMS), 1-1 Namiki, Tsukuba, Ibaraki 305-0044 Japan

[3]Research & Development Department, Corporate Photoreceptor Division,

KYOCERA Document Solutions Inc., 1-2-28 Tamatsukuri, Chuo-ku, Osaka 540-8585, Japan

[4]Institute of Pure and Applied Sciences, University of Tsukuba, 1-1-1 Tennodai, Tsukuba 305-8573, Ibaraki, Japan

[*]Corresponding authors E-mail address: ISLAM.Ashraful@nims.go.jp

Synthesis of C21 and C22 SAMs:

Synthesis route:

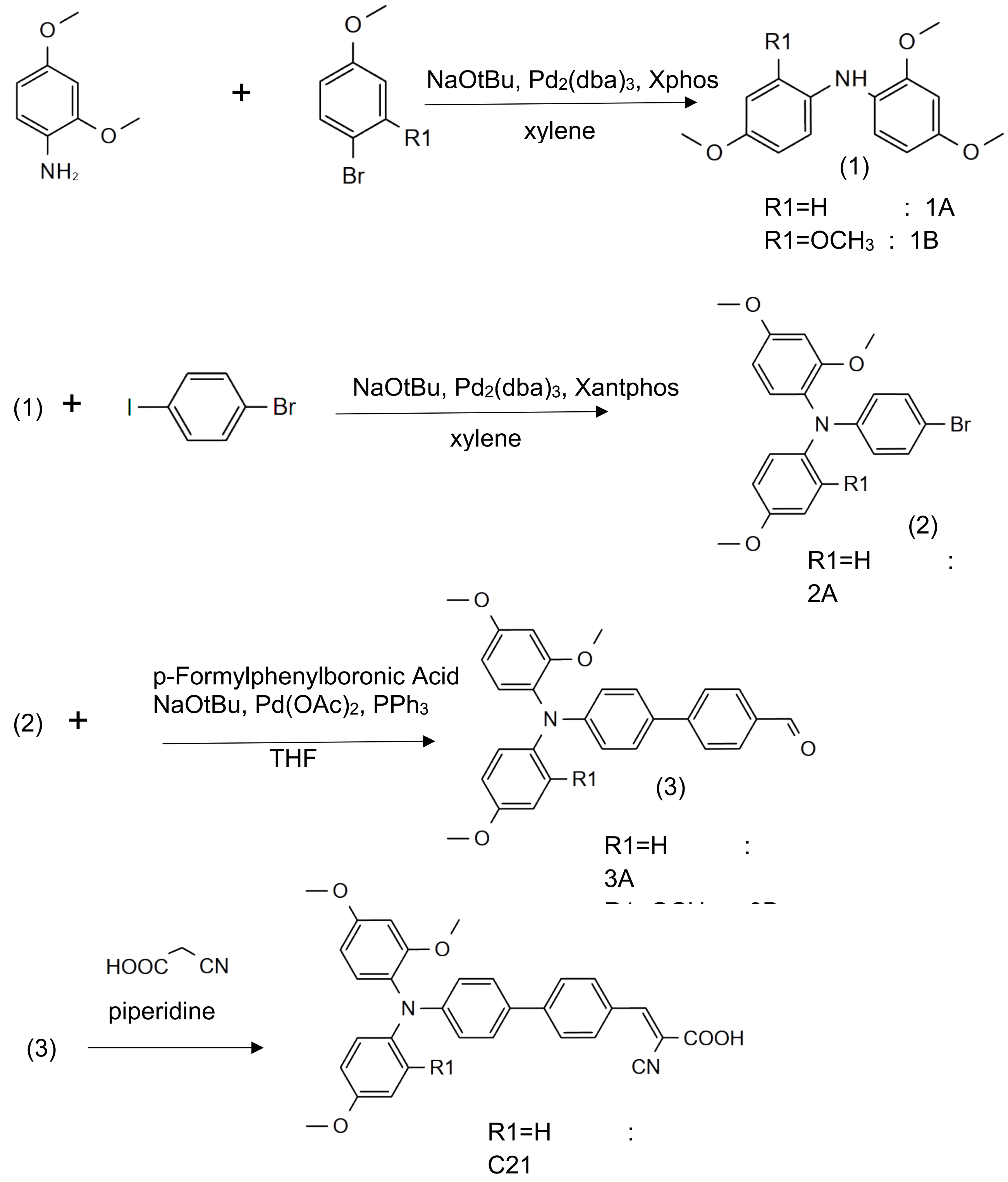

Scheme S1: Synthetic routes for SAMs.

Synthesis of 1A:

Charge a 500 mL two-neck flask fitted with a reflux condenser and a stir bar with 4-bromoanisole (20.57 g, 0.11 mol), 2,4-dimethoxyaniline (18.38 g, 0.12 mol), $Pd_2(dba)_3$ (tris(dibenzylideneacetone)dipalladium(0); 0.458 g, 0.5 mmol), XPhos (2-dicyclohexylphosphino-2',4',6'-triisopropylbiphenyl; 0.953 g, 2.0 mmol), and sodium tert-butoxide (11.53 g, 0.12 mol); purge with nitrogen. Add xylene (400 mL), heat to 100 °C with stirring, and stir for 3.5 hours. After the reaction, treat with activated clay (bleaching earth), filter, concentrate on a rotary evaporator, and purify by silica gel column chromatography (toluene:isohexane = 1:1, v/v). Concentrate and dry under vacuum at 70 °C to obtain Intermediate 1A as a reddish-brown oil (yield: 24.20 g, 84.8%; purity: 99.7%).

Synthesis of 2A:

Charge a 500 mL two-neck flask fitted with a reflux condenser and a stir bar with Intermediate 1A (9.076 g, 0.035 mol), 1-bromo-4-iodobenzene (10.10 g, 0.0357 mol), $Pd_2(dba)_3$ (0.037 g, 0.04 mmol), Xantphos (4,5-bis(diphenylphosphino)-9,9-dimethylxanthene; 0.093 g, 0.16 mmol), and sodium tert-butoxide (4.373 g, 0.0455 mol); purge with nitrogen. Add xylene (70 mL), heat to 130 °C with stirring, and react for 5 hours. Treat once with activated clay, filter, concentrate under reduced pressure, and purify by silica gel column chromatography (toluene:isohexane = 1:2, v/v). Concentrate to obtain Intermediate 2A as a pale yellow clear oil (yield: 12.20 g, 84.1%; HPLC purity: 97.7%).

Synthesis of 3A:

Charge a 500 mL two-neck flask fitted with a reflux condenser and a stir bar with Intermediate 2A (3.314 g, 0.008 mol), 4-formylphenylboronic acid (1.694 g, 0.0113 mol), palladium(II) acetate (0.180 g, 0.8 mmol), and triphenylphosphine (0.839 g, 3.2 mmol); purge with nitrogen. Prepare 2.0 M aqueous K_2CO_3 by dissolving potassium carbonate (7.366 g, 0.0533 mol) in ion-exchanged water (26.65 mL). Add this solution and THF (130 mL) to the flask. Heat to 80 °C with stirring and react for 3 hours. After the reaction, allow phase separation, decant the organic layer, filter, and collect the filtrate. Add activated clay (10 g) and anhydrous Na_2SO_4 (20 g), stir at 80 °C for 10 minutes, cool, filter, and concentrate. Purify by silica gel column

chromatography (toluene:THF = 98:2, v/v) and concentrate to obtain Intermediate 3A as a pale orange clear oil (yield: 3.40 g, 96.7%; HPLC purity: 98.1%).

Synthesis of C21:

Charge a 500 mL two-neck flask fitted with a reflux condenser and a stir bar with Intermediate 3A (2.285 g, 0.0052 mol) and cyanoacetic acid (4.423 g, 0.052 mol); purge with nitrogen. Add piperidine (1.397 g, 0.0164 mol) and acetonitrile (65 mL), then reflux with stirring for 6 hours. Perform hot filtration, concentrate the filtrate under reduced pressure, and dry under vacuum to obtain a dark brown solid. Purify by silica gel column chromatography (toluene:THF = 1:1, v/v), concentrate, then precipitate by addition of toluene and isohexane. Collect by filtration to obtain C21 as a reddish-brown solid (yield: 0.79 g, 30%; HPLC purity: 99.1%).

^{1}H NMR (600 MHz, DMSO-d_6, δ, ppm): 8.30 (s, 1H), 8.06 (d, J = 8.3 Hz, 2H), 7.79 (d, J = 8.3 Hz, 2H), 7.57 (d, J = 8.9 Hz, 2H), 7.14–7.16 (m, 3H), 6.91 (d, J = 8.9 Hz, 2H), 6.69 (d, J = 2.8 Hz, 1H), 6.57 (d, J = 9.6 Hz, 3H), 3.78 (s, 3H), 3.73 (s, 3H), 3.69 (s, 3H).

Synthesis of 1B:

Prepared as for 1A using 1-bromo-2,4-dimethoxybenzene in place of 4-bromoanisole. 1B was obtained as a reddish-brown oil (yield: 23.90 g, 75.1%; purity: 97.7%).

Synthesis of 2B:

Prepared as for 2A using Intermediate 1B in place of 1A. 2B was obtained as a pale yellow clear oil (yield: 2.27 g, 14.6%; HPLC 98.3%; repeat: 2.35 g, 14.8%; HPLC 98.0%).

Synthesis of 3B:

Prepared as for 3A using Intermediate 2B in place of 2A. 3B was obtained as a pale orange clear oil (yield: 1.60 g, 42.6%; HPLC purity: 97.2%).

Synthesis of C22:

Prepared as for C21 using Intermediate 3B in place of 3A. C22 was obtained as a reddish-brown solid (yield: 0.89 g, 31.9%; HPLC purity: 98.7%).

^{1}H NMR (600 MHz, DMSO-d_6, δ, ppm): 8.29 (s, 1H), 8.05 (d, J = 8.3 Hz, 2H), 7.77 (d, J = 8.2 Hz, 2H), 7.52 (d, J = 8.9 Hz, 2H), 7.21 (d, J = 8.9 Hz, 2H), 6.68 (d, J = 2.8 Hz, 2H), 6.54 (dd, J = 8.2, 2.8 Hz, 4H), 6.29 (d, J = 8.9 Hz, 2H), 3.77 (s, 6H), 3.74 (s, 6H).

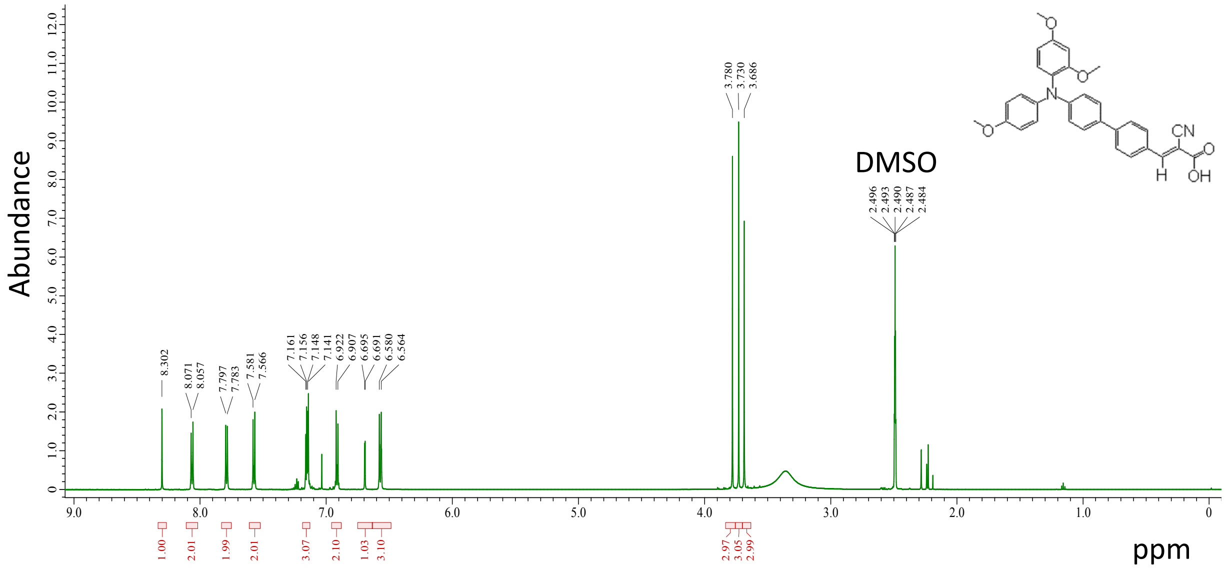

Figure S1. ^{1}H NMR spectra of C21 in DMSO-d_6.

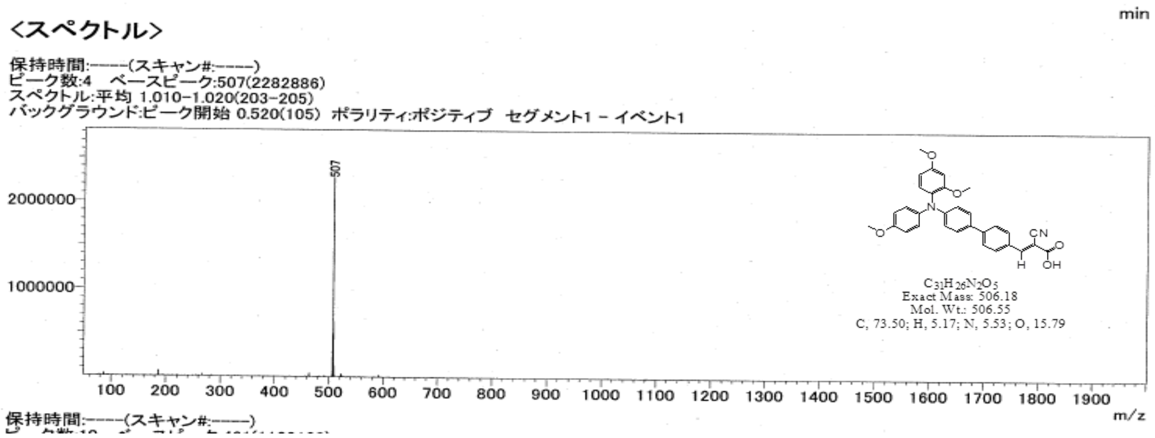

Figure S2. DART-MS spectra of C21.

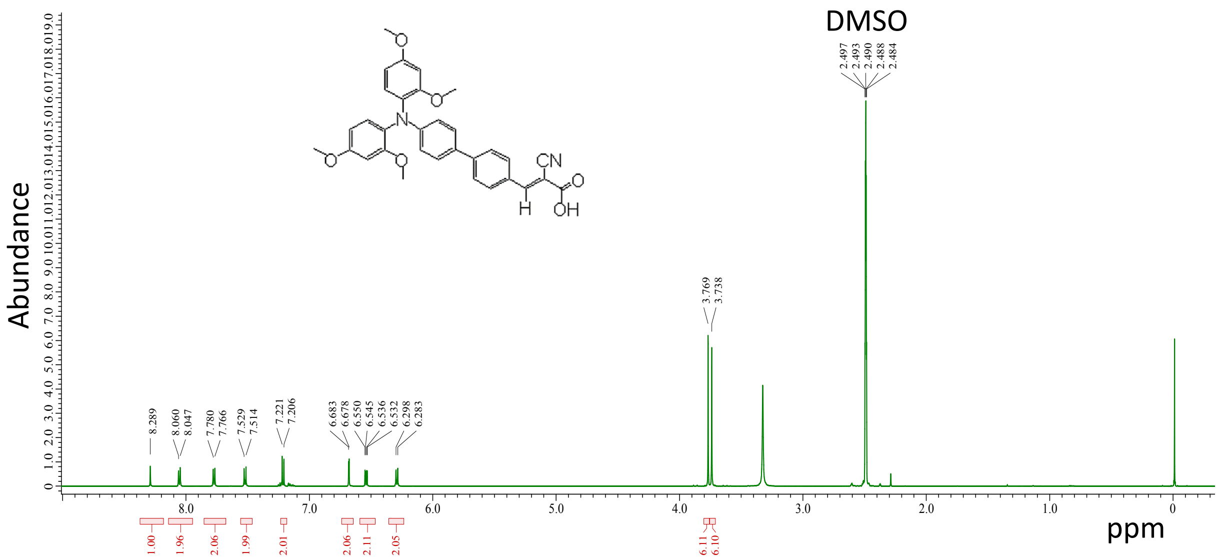

Figure S3. ^{1}H NMR spectra of C22 in DMSO-d_6.

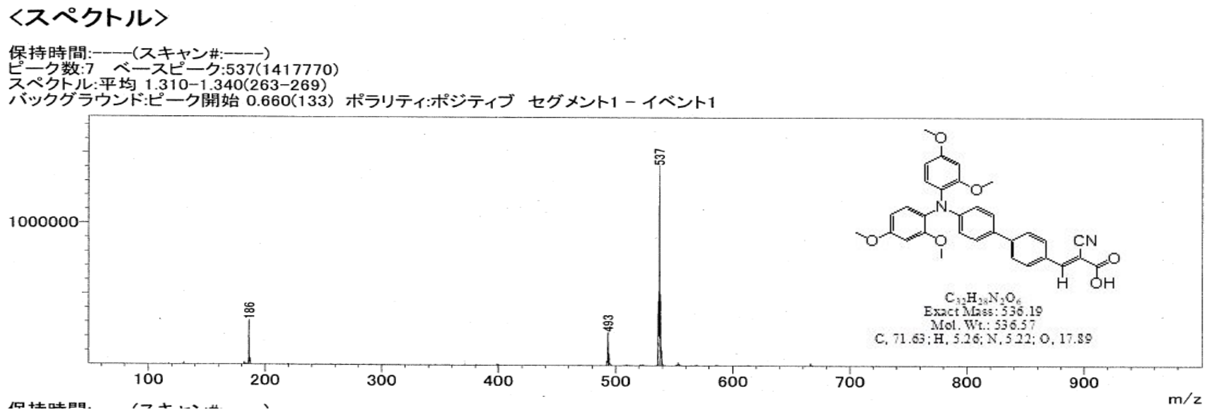

Figure S4. DART-MS spectra of C22.

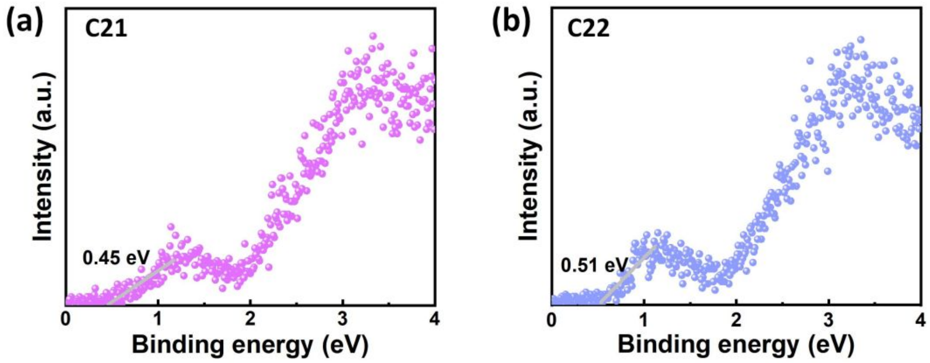

Figure S5. UPS spectra of the (a) FTO/C21 and (b) FTO/C22 in the valence band region.

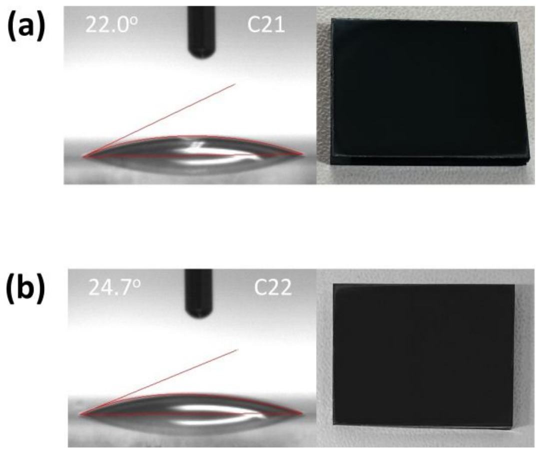

Figure S6. (a) WCA on C21 deposited on FTO and optical image of perovskite deposited on C21. (b) WCA on C22 deposited on FTO and optical image of perovskite deposited on C22.

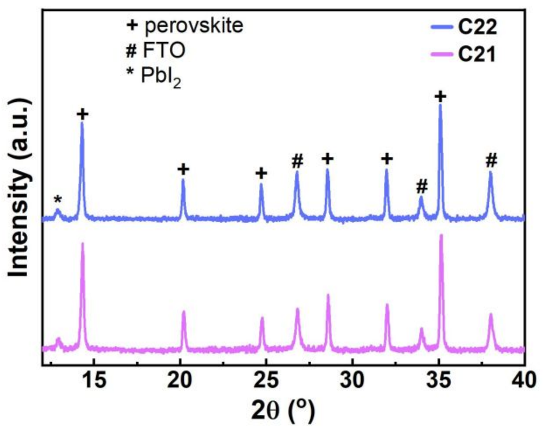

Figure S7. XRD analysis of perovskite on C21 and C22 SAMs.

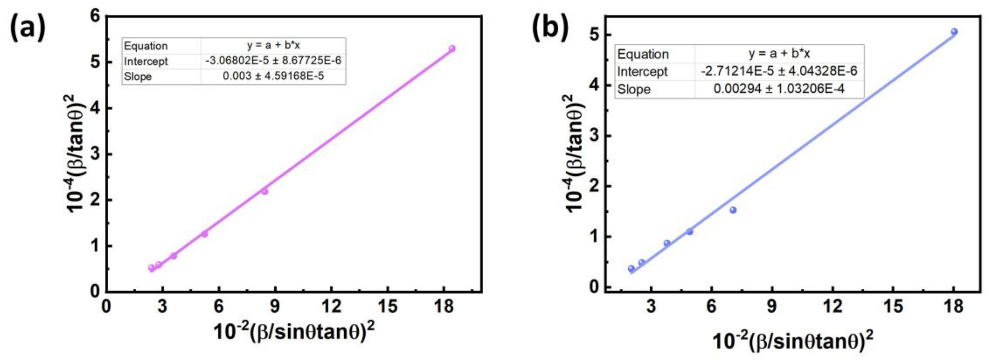

Figure S8. Halder–Wagner plots of perovskite deposited on (a) C21 and (b) C22 SAMs.

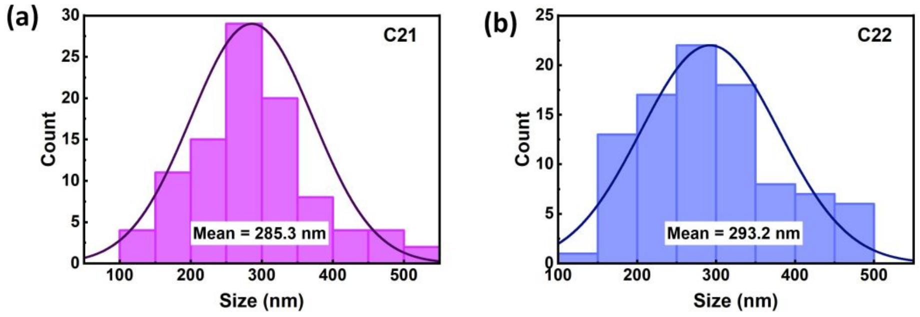

Figure S9. Grain size distribution of perovskite deposited on (a) C21 and (b) C22 SAMs.

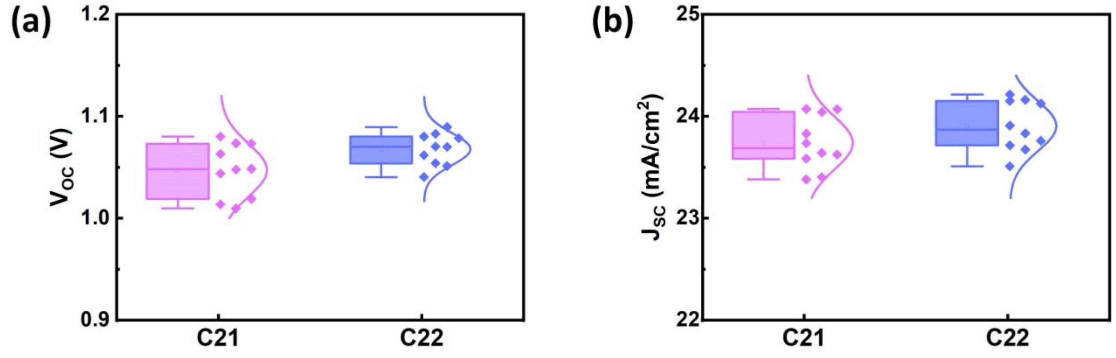

Figure S10. Statistical distribution of the (a) V_{OC} and (b) J_{SC} of the C21 and C22 -based PSCs under reverse scan.

Table S1. Latest performance of the perovskite solar cells with different terminal groups functionalized with different functional groups-based self-assembled monolayers (SAMs).

Functional group	SAM molecule	PCE	Reference
Electron-donating units<br>-OCH_3<br>-SCH_3	MeS−CbzPh	26.01%	*Angew. Chem. Int. Ed.* **2025**, *64*, e202419375
	MPA-CPA	25.16%	*Science* **2023**, *380*,404-409.
	MPA-Ph-CA	22.53%	*ACS Materials Letters* **2022**, *4*, 1976−1983.
	C21	21.10%	**This work**
	C22	21.58%	
Halogen units	DMAcPA	23.03%	*Nature* **2023**, *620*, 545–551.
	2BrPXZPA	22.98%	*Nanoscale* **2023**, *15*, 1676-1686.
Extending conjugation	Bz-PhpPACz	26.46%	*Nature Communication* **2025**, *16*, 86.
	IDCz-3	25.15%	*Advanced Materials* **2024**, *36*, 2401537.

Table S2. Summary of the deconvoluted peaks of C 1s of the C21 and C22 SAMs deposited on FTO substrate.

Peak	Binding energy (eV)		Peak area (%)	
	C21	C22	C21	C22
C-C	284	284.2	59.51	52.89
C-N	285.2	285.4	20.84	26.87
C-O-C	286.1	286.4	18.29	17.81
O-C=O	289.7	289.7	1.36	2.43

Table S3. Calculated crystallographic properties of perovskite deposited on C21 and C22 SAMs from the Halder–Wagner plot.

HTL	D (nm)	$\varepsilon \times 10^{-3}$
C21	46	1.38
C22	47	1.31

$$\left(\frac{\beta}{\tan\theta}\right)^2 = \frac{k\lambda}{D}\frac{\beta}{\tan\theta\sin\theta} + 16\varepsilon^2 \quad (S1)$$

$$\text{slope} = \frac{k\lambda}{D} \quad (S2)$$

$$\text{intercept} = 16\varepsilon^2 \quad (S3)$$

Where,

θ = Bragg angle

β = full width half maximum

ε = strain

D = crystallite size
